# *Lactobacillus johnsonii*-derived extracellular vesicles modulate gut microbiota metabolites and macrophage-related immune responses against *Salmonella* Typhimurium infection

**DOI:** 10.1186/s13567-026-01750-w

**Published:** 2026-04-29

**Authors:** Yi Wu, Keyuan Chen, Ting Hu, Yaoyao Luo, Yue Zhang, Guiyan Yang, Jiufeng Wang, Yaohong Zhu

**Affiliations:** 1 State Key Laboratory of Veterinary Public Health and Safety, College of Veterinary Medicine, China Agricultural University, Beijing, 100193 China; 2 China-Australia Joint Laboratory for Animal Health Big Data Analytics, College of Animal Science and Technology & Veterinary Medicine, Zhejiang A&F University, Hangzhou, 311300 China; 3 Beijing Center for Animal Disease Prevention and Control, Beijing, 102629 China

**Keywords:** Extracellular vesicles, *Salmonella* Typhimurium, gut microbiota, intestinal inflammation, intestinal microbiota metabolism

## Abstract

*Salmonella* Typhimurium is a significant pathogen causing diarrhea in weaned piglets, capable of triggering systemic inflammation and severe intestinal damage. As bioactive components of gut microbiota, extracellular vesicles (EVs) derived from *Lactobacillus johnsonii* (Lj-EVs) are likely to penetrate the intestinal mucus layer and reach host cells, potentially mediating host-microbiome interactions. Here, we found that Lj-EVs improved the intestinal barrier and attenuated intestinal injury in mice infected with *S.* Typhimurium. Multi-omics analysis showed that Lj-EVs intervention enriched mucin-utilizing *Akkermansia*. Moreover, Lj-EVs were associated with metabolic alterations, characterized by enhanced oxidative phosphorylation during homeostasis and suppression of HIF-1α-mediated glycolysis during infection. Proteomic profiling showed that Lj-EVs enriched functional proteins related to oxidative phosphorylation, indicating their potential immunomodulatory role. Notably, Lj-EVs modulated macrophage-mediated immune responses, contributing to limiting bacterial dissemination and alleviating intestinal inflammation. In conclusion, our study demonstrates that Lj-EVs alleviate *Salmonella* infection by reducing bacterial burden and confer metabolic and immunomodulatory benefits. These findings provide new evidence for future investigation of the protective roles of probiotic vesicles.

**Supplementary Information:**

The online version contains supplementary material available at 10.1186/s13567-026-01750-w.

## Introduction

The homeostasis of the intestinal microbiota is maintained through dynamic information exchange between microorganisms and the host [1]. As beneficial members of microbiota, probiotics contribute to intestinal homeostasis by reshaping microbial diversity, antagonizing pathogen colonization, and regulating host immune responses [2]. Notably, this cross-boundary communication often occurs not through direct contact between microbes and host cells, but via microbially secreted derivatives [3, 4]. Among these, extracellular vesicles (EVs) with lipid bilayer structures have garnered increasing attention as key mediators of intercellular communication [5]. EVs are capable of carrying active cargo (including proteins, nucleic acids, and metabolites) across the intestinal mucus layer to reach host cells, thereby facilitating host-microbiome interactions [6].

Based on the delivery advantages and metabolic regulatory potential of EVs, emerging evidence highlights the role of probiotic-derived EVs in regulating intestinal inflammation through multimodal mechanisms [7–9]. For example, EVs from the commensal bacterium *Bacteroides vulgatus* mpk could deliver microbial ligands to immune cells and participate in host immune responses [7]. *Lactobacillus acidophilus*-derived membrane vesicles deliver antibacterial agents that inhibit opportunistic pathogen and reshape microbial community composition [8]. Similarly, *Lactobacillus plantarum*-derived extracellular vesicles could modulate macrophage polarization and the lysine degradation pathway for alleviating ulcerative colitis [9]. Collectively, these findings underscore the unique value of EVs as nanoscale messengers within the intestinal micro-environment.

Metabolic competition between pathogen and host represents a central battlefield in intestinal infections [10]. *Salmonella* Typhimurium, a classic invasive intestinal pathogen, employs virulence strategies centered on disrupting the intestinal barrier and hijacking the metabolic programs of host immune cells to facilitate systemic dissemination [11, 12]. Upon invasion, *Salmonella* utilizes its type III secretion system (T3SS) to penetrate resident macrophages in the intestinal lamina propria and subsequently rewires the host metabolic network to evade immune clearance [11, 13]. This infection triggers reprogramming of host cell metabolism and inflammatory responses, characterized by up-regulated glycolysis and a suppressed tricarboxylic acid cycle [14]. Such metabolic shifts are closely linked to macrophage polarization [15]. Specifically, macrophages undergo dynamic metabolic reprogramming to regulate their immune functions: the pro-inflammatory M1 phenotype relies on glycolysis to rapidly generate energy and release inflammatory mediators (e.g., TNF-α, IL-1β, iNOS), whereas the anti-inflammatory M2 phenotype relies on mitochondrial oxidative phosphorylation to support tissue repair. This phenotypic transition is largely driven by HIF-1α-mediated metabolic reprogramming [16]. Therefore, attenuating glycolysis-mediated M1 macrophage polarization presents a promising strategy to control *Salmonella* infection and maintain intestinal health.

Our previous study found that a probiotic strain of *Lactobacillus johnsonii* (L531) could attenuate *Salmonella*-induced diarrhea and intestinal inflammation in piglets by regulating the Th17/Treg balance [17, 18]. Since the strain-specific characteristics of probiotic bacteria, the effects of *L. johnsonii* L531-derived extracellular vesicles (Lj-EVs) on modulating host-pathogen interactions in response to *Salmonella* infection remain to be elucidated. This study investigates the hypothesis that Lj-EVs orchestrate a multidimensional regulatory network encompassing the gut microbiota, metabolism, and immunity. Specifically, Lj-EVs could ameliorate intestinal inflammation by inhibiting the HIF-1α/glycolysis axis activated by *S*. Typhimurium and drive a shift in macrophage polarization from M1 toward M2 phenotype. These results provide insights into how probiotics and their EVs influence gut health and offer strategies for the prevention of *Salmonella* infection.

## Materials and methods

### Bacterial strains and growth conditions

*Salmonella* Typhimurium (ATCC14028) used in this study was obtained from American Type Culture Collection. *S*. Typhimurium was grown in Luria–Bertani (LB, Land Bridge, Beijing, China) medium overnight at 37 ℃ with shaking at 200 rpm. Bacterial growth was monitored by measuring the optical density at 600 nm using a microplate reader (Epoch 2, BioTek Instruments, Winooski, VT, USA). *S*. Typhimurium was inoculated in fresh LB broth (1% volume ratio) at 37 ℃ until the OD_600_ achieved 0.6 (logarithmic growth phase).

*Lactobacillus johnsonii* (L531), previously isolated from the intestinal contents of healthy weaned piglets [17–19], was inoculated on De Man, Rogosa, and Sharpe (MRS, Land Bridge, Beijing, China) agar and incubated for 36 h at 37 ℃, and then subcultured in fresh medium. To maintain anaerobic conditions, all procedures were performed in an anaerobic chamber (Electron Corporation, La Calamine, Belgium).

### Preparation and characterization of *Lactobacillus johnsonii-*derived EVs

Bacteria were cultured in MRS broth for 36 h and then subcultured for another 18 h to early stationary phase before being used for supernatant fractionation. Bacterial growth was monitored by measuring the optical density (OD_600_ nm). Extracellular vesicles derived from *Lactobacillus johnsonii* (Lj-EVs) were isolated using differential ultracentrifugation according to established protocols [20, 21]. In short, *L. johnsonii* grown cultures were centrifuged at 5000 × *g* for 30 min at 4 ℃ to collect bacterial supernatants. It was then filtered through 0.22 μm-pore size cellulose acetate membrane (Merck Millipore, Darmstadt, Germany) and concentrated using Amicon Ultra −0.5 centrifugal filter units with a 100 kDa molecular weight cutoff (Ultracel-PL regenerated cellulose; Merck Millipore, Darmstadt, Germany) according to the manufacturer’s instructions. Concentrated supernatant was centrifuged at 150 000 × *g* for 90 min at 4 ℃ using an Optima XPN-100 ultracentrifuge equipped with a Type 70 Ti fixed-angle rotor (Beckman Coulter, Brea, CA, USA). Washed once with phosphate buffer solution (PBS), and centrifuged again under the same conditions for another 90 min. The sediments containing Lj-EVs were resuspended in PBS and stored at −80 ℃ for further experiments. The size distribution and concentration of Lj-EVs were analyzed by nanoparticle tracking analysis (NTA) using a NanoSight NS300 system (Malvern Panalytical, Malvern, UK). The morphology of the Lj-EVs was observed under an transmission electron microscope (Hitachi, Tokyo, Japan). All procedures adhered to the MISEV2023 guidelines for extracellular vesicle research [22].

### Cell culture

IPEC-J2 cells and Caco-2 cells (stored in our laboratory) were grown in DME/F-12 1:1 (Cytiva, Marlborough, MA, USA) and supplemented with 10% fetal bovine serum and 1% penicillin/streptomycin (ThermoFish Scientific, Waltham, MA, USA) at 37 ℃ in a CO_2_ incubator with 5% CO_2_ and a humidified (95%) atmosphere. RAW264.7 cells were obtained from the Cell Bank of the Chinese Academy of Sciences (Shanghai, China). The RAW 264.7 cell lines were cultured in DMEM containing 10% fetal bovine serum and 1% penicillin–streptomycin. Each cell line was authenticated by morphology and growth characteristics as well as manufacturing companies, and was assessed for mycoplasma contamination regularly.

### Flow cytometric analysis

For RAW264.7 cells, a constant panel of antibodies was used for consistency. The panel included antibodies against CD86-APC (610,330, BD Biosciences, San Jose, CA, USA), CD206-PE (17–2061-82, Thermo Fisher Scientific, San Diego, CA, USA), and F4/80-FITC (565,410, BD Biosciences, San Jose, CA, USA). During the incubation, the cell surface was stained with the antibody mixture for 30 min at 4 ℃. For intracellular staining of transcription factors, cells were stained for surface markers and fixed in Fix/Perm buffer (eBioscience, San Diego, CA, USA) for 30–60 min at RT, and permeabilized in permeabilization buffer (eBioscience, San Diego, CA, USA) at RT for 30 min in the presence of antibodies. Cells were acquired with a BD BioSciences^®^ LSRFortessa (BD Biosciences, San Jose, CA, USA), and analysis was performed with Kaluza^®^ Analysis Software (version 2.1; Beckman Coulter, Brea, CA, USA). The concentration, clone, and source of antibodies were kept constant to ensure consistency in staining. The analysis was completed using FlowJo software (Version 7.6.1, Treestar, Ashland, OR, USA).

### Distribution and internalization of Lj-EVs

To observe the biodistribution and internalization of Lj-EVs, Lj-EVs at 1.0 mg/mL were labeled with either DiR dye (Umibio, Shanghai, China) or PKH26 dye (Umibio, Shanghai, China) at concentrations of 5 μM each, and incubated at 37 ℃ for 30 min. 5 mL PBS was added to the incubated Lj-EVs-dye complex and homogenized. The free working solution was removed by centrifugation at 120 000 × *g* for 90 min to obtain purified and stained Lj-EVs. DiR-labeled Lj-EVs were administered orally to the mice. After mice were anesthetized, IVIS Spectrum imaging (PerkinElmer, Waltham, MA, USA) was performed at 0, 6, 12, 24, 48, 72 h, and the fluorescence intensity was observed. Imaging protocols were adapted from previously described methods [23]. PKH26-labeled Lj-EVs were co-cultured with RAW264.7 cells and *S.* Typhimurium, respectively, to observe the internalization of Lj-EVs in cells and bacteria using laser scanning confocal microscope (Leica Microsystems, Wetzlar, Germany).

### Animals and experimental design

C57BL/6 mice (6 weeks old, half male and half female) were obtained from Charles River Laboratory Animal Technology Co., Ltd. (Beijing, China). Mice were housed (no more than four per cage) in a pathogen-free animal facility under a 12 h light/12 h dark cycle and were provided free access to food and water ad libitum throughout the entire experiment. Before acclimatization, mice were matched for body mass and randomly divided into 6 groups (*n* = 8 per group): the Control group (CN), *L. johnsonii* control group (LC), Lj-EVs control group (EV), *S.* Typhimurium infection group (ST), *L. johnsonii* treatment group (LS), and Lj-EVs treatment group (ES). At the end of the experiments, the animals were anesthetized with isoflurane and blood samples were collected by retro-orbital sinus puncture, then the mice were killed by cervical dislocation. All experimental procedures were performed in accordance with the relevant guidelines and regulations and were approved by the Animal Ethics Committee of the China Agricultural University (Permit Number: AW41113202-2–1).

### *S*. Typhimurium infection in vivo

Before *S*. Typhimurium challenge, mice in LC and LS groups were orally administered with 0.2 mL PBS of *L. johnsonii* L531 (1 × 10^8^ CFU/mL) for 7 days; mice in EV and ES groups were orally administered with Lj-EVs (0.12 mg/mL) for 7 days; mice in CN and ST groups were administered an equal volume of PBS daily. On Day 8, the mice in ST, LS and ES groups were given 0.2 mL of *S*. Typhimurium (1 × 10^7^ CFU/mL) by gavage, whereas 0.2 mL PBS were orally administered to mice in CN, LC and EV groups. Blood samples were collected 3 days after infection, mice were then euthanized, and the liver, spleen, jejunum, ileum, and colon tissues were collected for histopathological examination. The feces as well as liver, jejunum, ileum and colon tissues were ground in sterile saline to quantify bacterial load.

### Assessment of diarrhea degree

The severity of diarrhea was evaluated using the dry/wet weight of fecal pellets. 0.5 g of fecal samples were collected and weighed. Then the feces were dried at 60 ℃ for 24 h until the weight change was less than 1%, and weighed again. The dry/wet weight ratio was calculated by comparing the dried weight to the initial weight.

### Fecal viable culture counts

The 0.1 g of feces was collected and homogenized (65 Hz) with 1 mL PBS using zirconia beads. The supernatant was separated from the homogenized suspension by centrifugation at 13 000 rpm for 15 min and placed at 4 ℃.

### Western blotting

Tissues were lysed using RIPA buffer (Solarbio, Beijing, China) containing a 1% protease/phosphatase inhibitor cocktail (Cell Signaling Technology, Danvers, MA, USA). Protein concentrations were determined with a BCA Protein Assay Kit (Beyotime Biotechnology, Inc., Shanghai, China). These protein lysates were separated by sodium dodecyl sulphate polyacrylamide gel electrophoresis (SDS-PAGE) and then transferred onto polyvinylidene fluoride (PVDF) membranes (Immobilon-P, Merck Millipore, Burlington, MA, USA). After being blocked with 5% Bovine Serum Albumin (BSA), the membranes were then incubated with primary antibodies at 4 ℃ overnight: anti-β-actin (1:1000, Proteintech, 66,009–1-Ig), anti-ɑ-tubulin (1:5000, Proteintech, 80,762–1-RR), anti-HIF-1ɑ (1:1000, Abcam, ab51608), anti-iNOS (1:1000, Proteintech, 22,226–1-AP), anti-PKM2 (1:1000, Selleck, F0295), anti-Arg1 (1:10 000, Proteintech, 16,001–1-AP) and anti-GLUT1 (1:5000, Proteintech, 21,829–1-AP). The secondary antibody HRP-labeled goat anti-rabbit IgG (H + L) (PR30009) and the HRP-labeled goat anti-mouse IgG (H + L) (HS201-01) were purchased from Proteintech (Rosemont, IL, USA). After incubation with corresponding secondary antibodies (1:5000) for 45 min at room temperature, ECL reagents (Bio-Rad, Hercules, CA, USA) were used to develop bands. Blots were imaged with a ChemiDoc MP Imaging System (Bio-Rad Laboratories, Hercules, CA, USA). The density of protein was quantified using the ImageJ (National Institutes of Health, Bethesda, MD, USA).

### Histological analysis

Fresh liver, spleen, jejunum and colon were fixed with 4% formaldehyde. The tissue was first embedded in paraffin blocks, followed by sectioned and stained with hematoxylin and eosin (H&E) staining. All slides were scanned under a light microscope using an Olympus BX53 optical microscope (Olympus Corporation, Tokyo, Japan). At least 3 fields of view per section were measured and the images were manually delineated and analyzed using ImageJ.

### Immunohistochemical staining

Jejunal and colic tissues were fixed in 4% formaldehyde overnight and were then embedded and implanted into paraffin blocks. After dewaxing and rehydration, the tissue sections were placed in citrate buffer (pH = 6) for antigen repair at 100 ℃ for 20 min. Block with 5% bovine serum albumin (BSA) at room temperature for 30 min, and then incubated overnight at 4 ℃ with the following primary antibodies: anti-Occludin (1:200, Proteintech, 27,260–1-AP), anti-MUC2 (1:200, Proteintech, 27,675–1-AP). Subsequently, a histochemical secondary antibody kit (PV6001; Beijing Zhongshan Jinqiao Biotechnology Co., Ltd., Beijing, China) was used for 1 h at room temperature. Sections were read using an Olympus CX31 optical microscope (Evident Scientific, Tokyo, Japan), and images were captured with OVT view 3.7 software (OVT Software Technology Co., Ltd., Jiangsu, China).

### Immunofluorescence

For tissue immunofluorescence staining, the jejunal and colic sections were blocked with 10% BSA and then incubated overnight at 4 ℃ with the following primary antibodies: anti-ZO-1 (1:500, Proteintech, 21,773–1-AP), anti-F4/80 (1:200, Thermo, 14–4801-82), anti-IRG1(1:1000, Abcam, ab222411), anti-CD206 (1:200, Abcam, ab64693), and anti-iNOS (1:200, Proteintech, 22226–1-AP). This was followed by incubation with the following secondary antibodies: donkey anti-mouse Alexa Fluor 488 (1:500, Abcam, ab150105), and goat anti-rabbit Alexa Fluor 647 (1:500, Abcam, ab150083). The Alexa Fluor 555-labeled secondary antibody (A0453) was purchased from Beyotime Biotechnology. The nuclei were then stained with 4′,6-diamidino-2-phenylindole (DAPI), and microphotographs were obtained using a confocal microscope (LSM710, Zeiss, Heidenheim, Germany). The average intensity for each field was measured by the ImageJ software.

### RNA extraction and qRT-PCR analysis

Total RNA was extracted from the jejunum tissues of animals using RNAiso Plus (Takara, SanJose, CA, USA) according to the manufacturer’s instructions. To obtain cDNA, reverse transcription reactions were performed using the PrimeScriptTM RT kit (RR047A, TaKaRa, Kyoto, Japan). Primer sequences used are listed in Table 1. qPCR reactions were performed using NovoStar SYBR qPCR SuperMix Plus (Novoprotein, E096-01A, China) in a 7500 real-time PCR system (Applied Biosystems, Inc., USA). The mRNA levels of target genes were normalized to the Glyceraldehyde-3-phosphate dehydrogenase (GAPDH) expression. Quantification of qPCR results was performed by the 2^−△△CT^ method.

### 16*S* rRNA gene sequencing and microbiota metabolomic analysis

The caecum of each mouse was collected in a sterile tube, immediately frozen in liquid nitrogen and stored at −80 ℃ until further use. DNA was extracted using the QiaAmp Fast DNA Stool Mini Kit (Qiagen, Hilden, Germany), with slight modification of the manufacturer’s protocol. Caecal samples were also homogenized with stainless steel beads (5 mm; Qiagen) after homogenization with glass beads (0.1 mm). Illumina-adapted universal primers 515F/806R, which target the V4 region of the 16*S* rRNA, were used for DNA amplification, and the DNA was then purified using the QIAquick PCR purification Kit (Qiagen, Hilden, Germany). Sequencing was performed on an Illumina MiSeq platform (Illumina, San Diego, CA, USA). Metabolites were extracted and analyzed using a Vanquish Neo UHPLC System coupled to a Q Exactive HF mass spectrometer (Thermo Fisher Scientific, Bremen, Germany).

### Proteolysis, LC–MS/MS and proteomics analysis of Lj-EVs

The analysis was performed using a shotgun proteomics approach based on liquid chromatography-high resolution tandem mass spectrometry. In brief, EVs were dissolved in 5 mM Dithiothreitol (DTT) and reduced at 56 ℃ for 30 min. Then iodoacetamide (IAA) was added to make its final concentration 11 mM, and incubated at room temperature for 15 min away from light. The alkylated samples were transferred to an ultrafiltration tube, centrifuged at 12 000 × *g* for 20 min at room temperature, replaced three times with 8 M urea, and then three times with displacement buffer, trypsin was added at a ratio of 1:50 (protease: protein, m/m), and digested enzymatically overnight. The peptides were recovered by centrifugation at 12 000  × *g* for 10 min at room temperature, then once by ddH_2_O, and the peptide solution was combined twice for later use. Peptides were dissolved by liquid chromatography mobile phase A and separated using a vanquish neo Ultra performance liquid system. The results obtained from proteomics analysis were derived from three repeated trials. Quality control analysis of peptide and protein levels was performed based on the search results of UniProt database.

### Statistical analysis

GraphPad Prism software (version 8.0.2; San Diego, CA, USA) was used for statistical analysis. Data were presented as the mean ± SD or as individual data points unless otherwise specified. The normality of the probability distribution was confirmed by the Shapiro Wilk test and the variance in the probability distribution was confirmed by the Brown-Forsythe test to determine the appropriate statistical analysis method using IBM SPSS statistics 23.0 (Chicago, IL, USA). Statistical comparisons between only two groups were conducted using unpaired Student’s two-tailed *t*-test (normal probability distribution and homogeneity of variance) or the Mann Whiney *U* test (abnormal probability distribution or heterogeneity of variance). For comparisons of more than two groups, a one-way analysis of variance (ANOVA) test (normal probability distribution) or non-parametric Kruskal Wallis test (abnormal distribution) followed by Dunn’s multiple comparison post hoc test. Additional detailed methods are provided in the corresponding figure legends. Statistically significance was indicated by asterisks as follows: ^*^P < 0.05; ^**^P < 0.01, ^***^P < 0.001, ^****^P < 0.0001.

## Results

### Isolation and characterization of *Lactobacillus johnsonii*-derived extracellular vesicles (Lj-EVs) and preliminary study on anti-inflammatory activity in vitro

**Figure 1:**
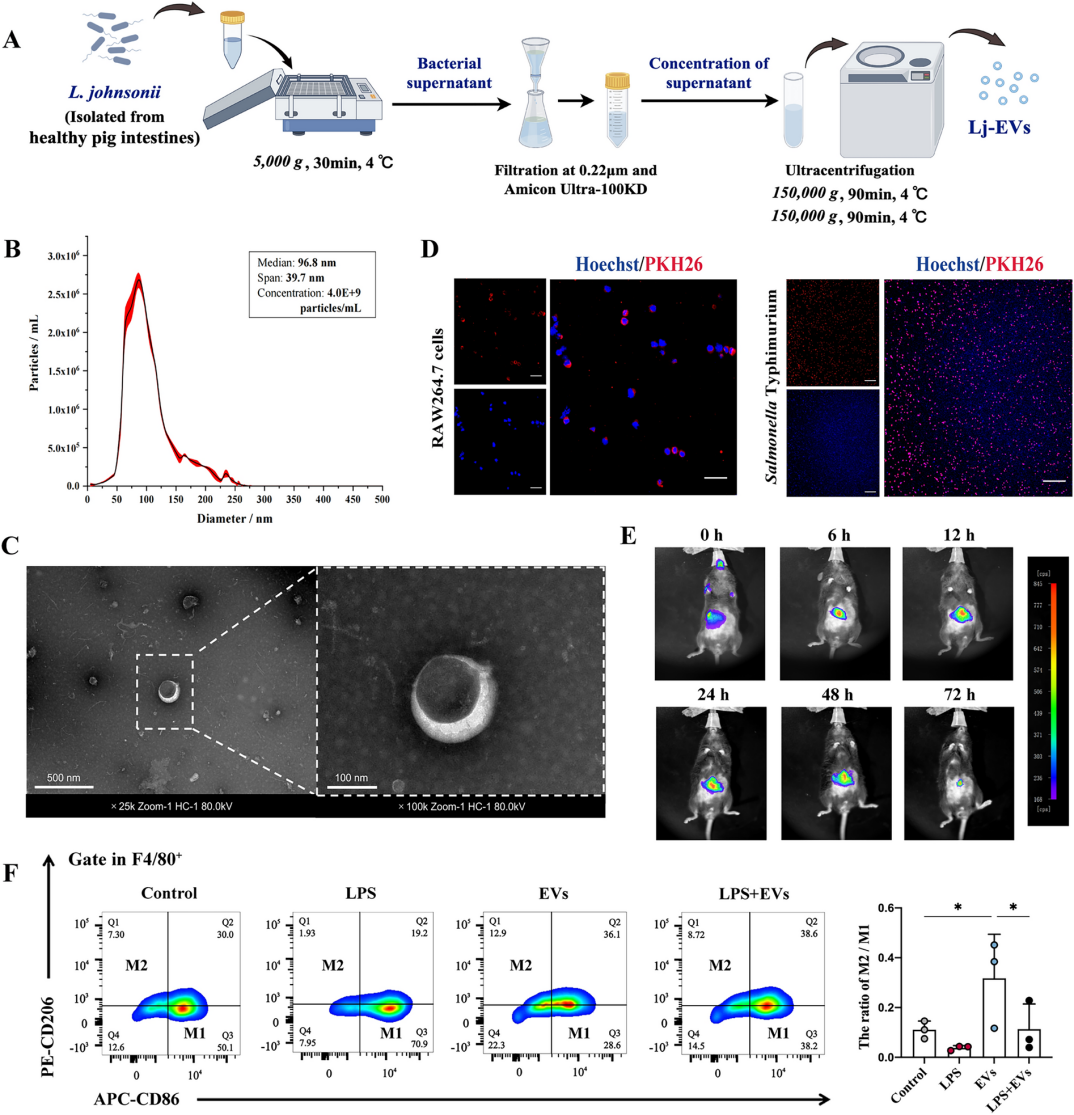
***Lactobacillus johnsonii*** **extracellular vesicles (Lj-EVs) isolation, characterization and in vitro anti-inflammatory activity.** (**A**) Illustration of the Lj-EVs isolation process. (**B**) Size distribution and concentration of Lj-EVs as measured in NanoSight. (**C**) Lj-EVs were characterized by transmission electron microscopy (TEM) as spherical membrane particles. Scale bars correspond to 500 nm (× 25 k) or 100 nm (× 100 k). (**D**) Uptake and internalization of Lj-EVs by RAW264.7 cells and *S.* Typhimurium. CLSM images of cells incubated with PKH26-labeled Lj-EVs for 2 h (left); bacteria incubated with PKH26-labeled Lj-EVs for 30 min (right). Scale bar = 50 μm. (**E**) Distribution of Lj-EVs after oral administration. C57BL/6 J mice were orally administered DiR-labeled Lj-EVs (50 ug/200 µL) and then visualized in the in vivo imaging system. Representative images depicted the tissue distribution of fluorescence signal in mice at 0, 6, 12, 24, 48 and 72 h after oral treatment of Lj-EVs. (**F**) Flow cytometry analysis of the proportion of CD206 and CD86 in F4/80 + RAW264.7 cells treated with Lj-EVs for 24 h with control or LPS (M1), respectively. Histogram is representative of three independent experiments (mean ± SD, ^*^P < 0.05). Figure  1 A was created with Figdraw.com under academic license.

Lj-EVs were obtained through a series of filtrations and ultra-high speed differential centrifugation (Figure 1A). Nanoparticle tracking analysis (NTA) of the purified Lj-EVs showed that the majority of particles fell within a size range of 50–150 nm (Figure 1B), which is consistent with the typical size distribution of bacterial EVs [5, 6]. The vesicular structure and lipid bilayer of the isolated particles were further confirmed by transmission electron microscopy (TEM) (Figure 1C). Then we fluorescently labeled the Lj-EVs using the lipophilic dye PKH26. The PKH26-labeled Lj-EVs were incubated with RAW 264.7 cells for 2 h and with *S*. Typhimurium for 30 min, respectively. CLSM images (Figure 1D) showed that the fluorescence of the Lj-EVs was localized around the nucleus of RAW264.7 cells and attached to a large number of *S.* Typhimurium. These results suggested that Lj-EVs could be taken up and internalized by cells and bacteria. To track the distribution of Lj-EVs in vivo, mice were orally gavaged with DiR-labeled Lj-EVs and monitored using biophoton imaging (Figure 1E). Lj-EVs-treated mice exhibited a stronger fluorescence signal localized to the gastrointestinal tract at 6 h post-administration. This fluorescence intensity within the gut progressively increased from 6 to 48 h, suggesting accumulation and persistence of Lj-EVs in the intestinal lumen. We then assessed the potential anti-inflammatory effects of Lj-EVs in cellular models (Additional file 1). In vitro treatment with Lj-EVs significantly attenuated the inflammatory responses in both LPS-stimulated RAW 264.7 cells and IPEC-J2 cells, as well as in Caco-2 cells infected with *S.* Typhimurium-infected (Additional file 1). Furthermore, flow cytometric analysis revealed that Lj-EVs treatment increased the M2/M1 ratio by up-regulating the expression of CD206 (an M2 macrophage-associated marker) and down-regulating the expression of CD86 (an M1 macrophage-associated marker) in RAW 264.7 cells (Figure 1E). These data indicated the potential of Lj-EVs to combat intestinal inflammation-related diseases and exerted immune function.

### Lj-EVs ameliorated *Salmonella* Typhimurium-induced infection and reduced bacterial burden in mice

**Figure 2:**
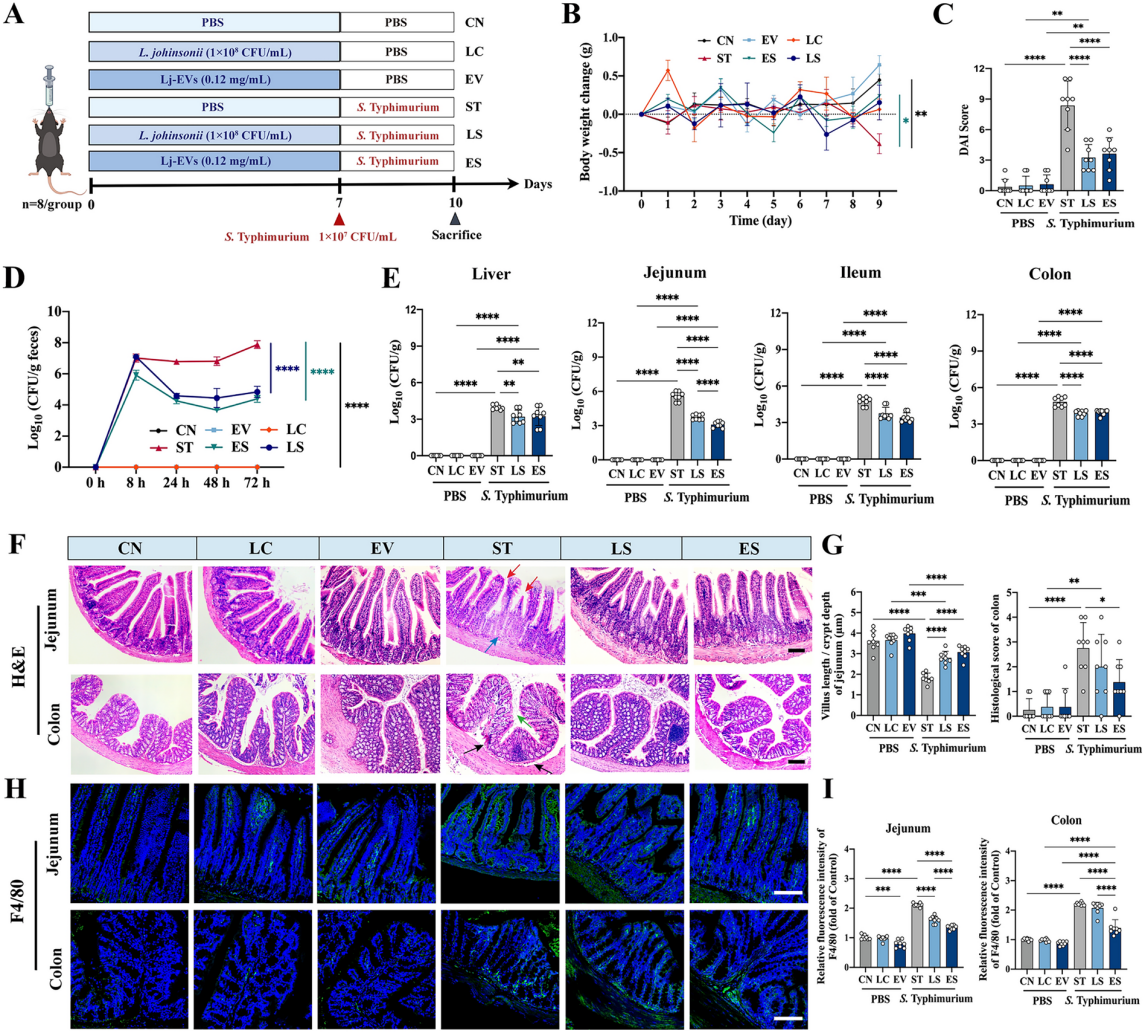
**Lj-EVs reduce the load of** ***Salmonella*** **Typhimurium in mice to alleviate intestinal infection and diarrhea.** (**A**) Experimental design. (**B**) Monitoring of body weight changes in mice expressed as the initial weight returned to zero. (**C**) Disease activity index (DAI) score was obtained at the end of the experiment, assessed by composite scoring of weight loss (0–4), fecal morphology (0–4), and intestinal bleeding (0–4). (**D**) Excretion of *Salmonella* in the feces of mice during 72 h post-infection, determined by serial dilution plating on XLT4 agar. (**E**) *S*. Typhimurium colonization levels in target organs (liver, jejunum, ileum and colon) at sacrifice. Tissues were homogenized and plated for CFU enumeration. (**F**) Representative H&E staining images of jejunum and colon. Red arrows indicate the villi, blue arrow indicates crypts, green arrow indicates intestinal glands, and black arrows indicate the basement membrane. Scale bars correspond to 400 μm. (**G**) Morphometric analysis of jejunal villus length to crypt depth ratio and semi-quantitative histoscoring (0–4 scale) of colon lesions assessing epithelial integrity, leukocyte infiltration, and edema. (**H**) Immunofluorescence quantification of F4/80 + macrophages (green) with DAPI nuclear counterstain (blue). Scale bars correspond to 100 μm. (**I**) Quantitative analysis of expression of F4/80 cells in intestinal sections by fluorescence intensity (ImageJ *v1.53*). Data represent mean ± SD in Figures 2B–E, G, I. *n* = 8. Statistical significance of Figure 2B, D was determined by two-way ANOVA Sidak’s multiple comparisons test; Statistical significance of Figures 2C, E, G, I was determined by one-way ANOVA with Tukey’s post-hoc test (^*^P < 0.05, ^**^P < 0.01, ^***^P < 0.001, ^****^P < 0.0001). Figure 2A was created with Figdraw.com under academic license.

To evaluate the protective role of Lj-EVs in vivo, we established a murine model of *S.* Typhimurium (ST)-induced intestinal inflammation (Figure 2A). Mice infected with *S*. Typhimurium exhibited a significant loss of body weight, a symptom that was markedly alleviated by pretreatment with Lj-EVs (*P* < 0.05 vs. ST group; Figure 2B). The clinical disease activity index (DAI) was also significantly lowered by both Lj-EVs and the parental *L. johnsonii* probiotics, with no statistically significant difference observed between these two treatment groups (Figure 2C). We next assessed the impact of Lj-EVs on bacterial colonization. Lj-EVs treatment significantly reduced *Salmonella* load in feces within 72 h post-infection (*P* < 0.01 vs. ST group; Figure 2D). At the endpoint of the experiment, the bacterial loads in liver, jejunum, ileum and colon were all significantly lower in the Lj-EVs-treated group (*P* < 0.01 vs. ST group; Figure 2E). Notably, Lj-EVs appeared more effective than intact probiotics in limiting bacterial colonization in the jejunum. *S*. Typhimurium infection induced severe diarrhea, as indicated by a reductive fecal dry-to-wet weight ratio (*P* < 0.01 vs. CN group) and watery stool consistency (Additional file 2A and B). Lj-EVs pretreatment restored near-normal stool consistency and significantly increased the fecal dry-to-wet ratio (*P* < 0.05 vs. ST group).

### Lj-EVs alleviated *Salmonella* Typhimurium-induced systemic inflammation and intestinal injury in mice

To evaluate the regulatory effects of Lj-EVs on systemic inflammatory responses, we first conducted histopathological analyses of the liver and spleen. In *S.* Typhimurium (ST)-infected mice, the liver exhibited extensive parenchymal necrosis, architectural disorganization, and severe inflammatory cell infiltration, accompanied by significantly elevated histopathological scores (*P* < 0.01 *vs.* CN group; Additional file 2). Remarkably, Lj-EVs substantially attenuated ST-induced hepatic damage, as evidenced by reduced inflammatory infiltration, preserved lobular architecture, and diminished necrotic areas (*P* < 0.01 vs. ST group). In spleen, ST infection caused disruption of tissue morphology, characterized by loss of demarcation between red pulp (RP) and white pulp (WP), lymphoid depletion in WP with stellate cell hyperplasia, and RP congestion due to erythrocyte accumulation (Additional file 2C-D). Lj-EVs pretreatment restored splenic architecture, with clearly defined RP-WP boundaries, reduced the area of splenic corpuscle (*P* < 0.01 *vs.* ST group), and normalized RP congestion. Notably, Lj-EVs increased the proportion of intact splenic follicles in uninfected mice without altering overall splenic structure, suggesting a potential role in maintaining lymphoid homeostasis. Immunofluorescence staining further demonstrated that Lj-EVs significantly suppressed macrophage infiltration (F4/80 + cells) in both liver and spleen tissues (*P* < 0.01 vs. ST group; Additional files 2E and F), suggesting that they may be involved in the control of systemic inflammation by regulating the migration of innate immune cells.

Based on the above findings, we next focused on the effects of Lj-EVs on the local inflammatory microenvironment of the intestines. As shown in Figure 2F, histopathological analysis revealed *S*. Typhimurium*-*induced intestinal damage. Jejunal villi were markedly shortened, edematous, and disorganized, with blunting and fusion of adjacent villi (red arrows), and the crypt structure appeared severe damage (blue arrow). Colonic tissues showed glandular architectural disruption and glandular cell exfoliation (green arrow), intestinal glands detached from basement membrane (black arrows), and lamina propria inflammatory infiltration. Lj-EVs attenuated *Salmonella* damage to the small intestine by increasing the height and orderly arrangement of jejunal villi and the depth of jejunal crypts (Figure 2G, left). In colon, Lj-EVs attenuated submucosal inflammatory cell infiltration, restored crypt morphology, and reduced glandular structure loss (*P* < 0.01 vs. ST group; Figure 2G, right). Given the critical role of macrophages in bacterial clearance [11], we quantified F4/80^+^ cell infiltration in the intestine. *S*. Typhimurium infection triggered massive macrophage recruitment in both jejunum and colon (*P* < 0.01 vs. CN group; Figure 2H). Lj-EVs treatment obviously suppressed macrophage infiltration in jejunal and colonic tissues (*P* < 0.01 vs. ST group; Figure 2I). These findings collectively indicated that Lj-EVs has a high potential to mitigate *Salmonella*-driven systemic and local inflammation by preserving tissue integrity and modulating innate immune cell recruitment.

### Lj-EVs improved intestinal inflammation induced by *Salmonella* Typhimurium infection and enhanced intestinal barrier integrity

**Figure 3:**
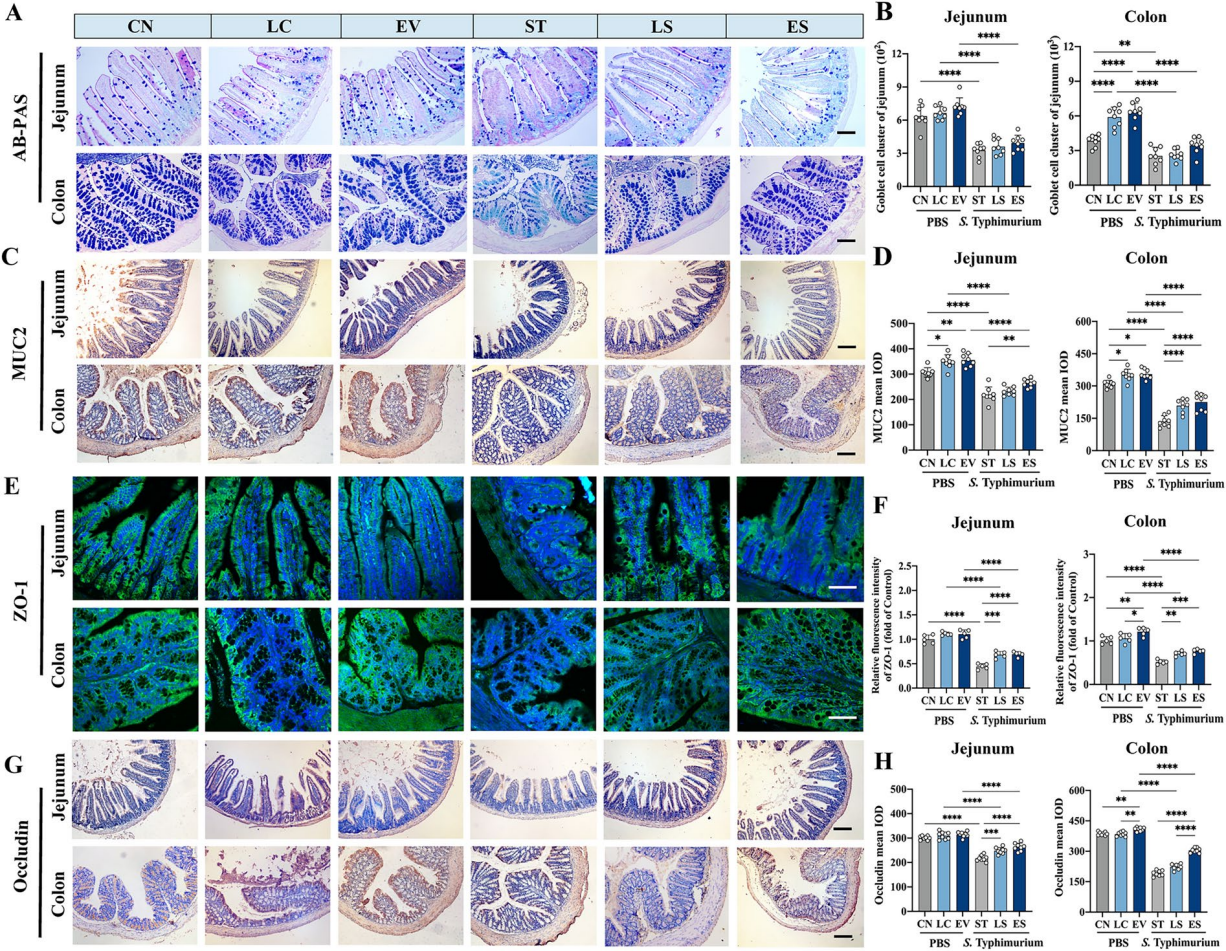
**Lj-EVs enhance the intestinal barrier to alleviate the inflammation of jejunum and colon caused by *Salmonella* Typhimurium infection in mice.** (**A**) Representative Alcian Blue-Periodic Acid Schiff (AB-PAS) staining images of jejunum and colon sections. Scale bars correspond to 400 μm. (**B**) Semi-quantitative analysis of goblet cell cluster of jejunum and colon using ImageJ *v1.53*. (**C**) MUC2 mucin localization in jejunum and colon of mice by immunohistochemistry (IHC) with DAB chromogen (brown). Counterstaining with hematoxylin (blue). Scale bars correspond to 400 μm. (**D**) Quantitative MUC2 expression using integrated optical density (IOD) measured by Image-Pro Plus v6.0 with background subtraction. (**E**) ZO-1 tight junction protein distribution visualized by immunofluorescence (green). Nuclei counterstained with DAPI (blue). Scale bars correspond to 100 μm. (**F**) ZO-1 fluorescence intensity quantification normalized to control group (CN). (**G**) Immunohistochemical analysis of Occludin expression in mouse jejunum and colon sections. Scale bars correspond to 400 μm. (**H**) IHC images showed the mean IOD of Occludin. Quantitative Occludin expression using integrated IOD measured by Image-Pro Plus v6.0 with background subtraction. Three random fields per sample were analyzed. Data present as means ± SD. Each dot represents data from individual biological replicate. Statistical significance of Fig. 3B, D, F, H determined by one-way ANOVA with Tukey’s post-hoc test (^*^P < 0.05, ^**^P < 0.01, ^***^P < 0.001, ^****^P < 0.0001).

We next systematically evaluated the multidimensional effects of Lj-EVs on intestinal barrier by examining mucus layer composition and epithelial tight junctions. Alcian blue-periodic acid-Schiff (AB-PAS) staining revealed that Lj-EVs pretreatment significantly increased goblet cell density in both jejunal and colonic tissues (*P* < 0.01 vs. ST group; Figures 3A–B), correlating with enhanced mucin secretion. Consistently, immunohistochemical analysis demonstrated that Lj-EVs significantly up-regulated MUC2 protein expression (the primary mucin glycoprotein) in the jejunum and colon (*P* < 0.01 vs. ST group; Figures 3C–D). Then we observed the expression of tight junction proteins that are crucial for epithelial barrier integrity. Immunofluorescence staining showed that Lj-EVs markedly enhanced ZO-1 expression at intercellular junctions, with fluorescence intensity increased in the jejunum and colon (*P* < 0.01 vs. ST group; Figures 3 E and F). Similarly, immunohistochemical quantification of Occludin revealed a clear increase in jejunal and colonic expression following Lj-EVs treatment (*P* < 0.01 vs. ST group; Figures 3G and H). These findings collectively indicated that Lj-EVs fortify intestinal barrier function by augmenting mucus layer production and reinforcing epithelial tight junctions.

### Lj-EVs reshaped the gut microbiota composition in *Salmonella* Typhimurium-infected mice

**Figure 4:**
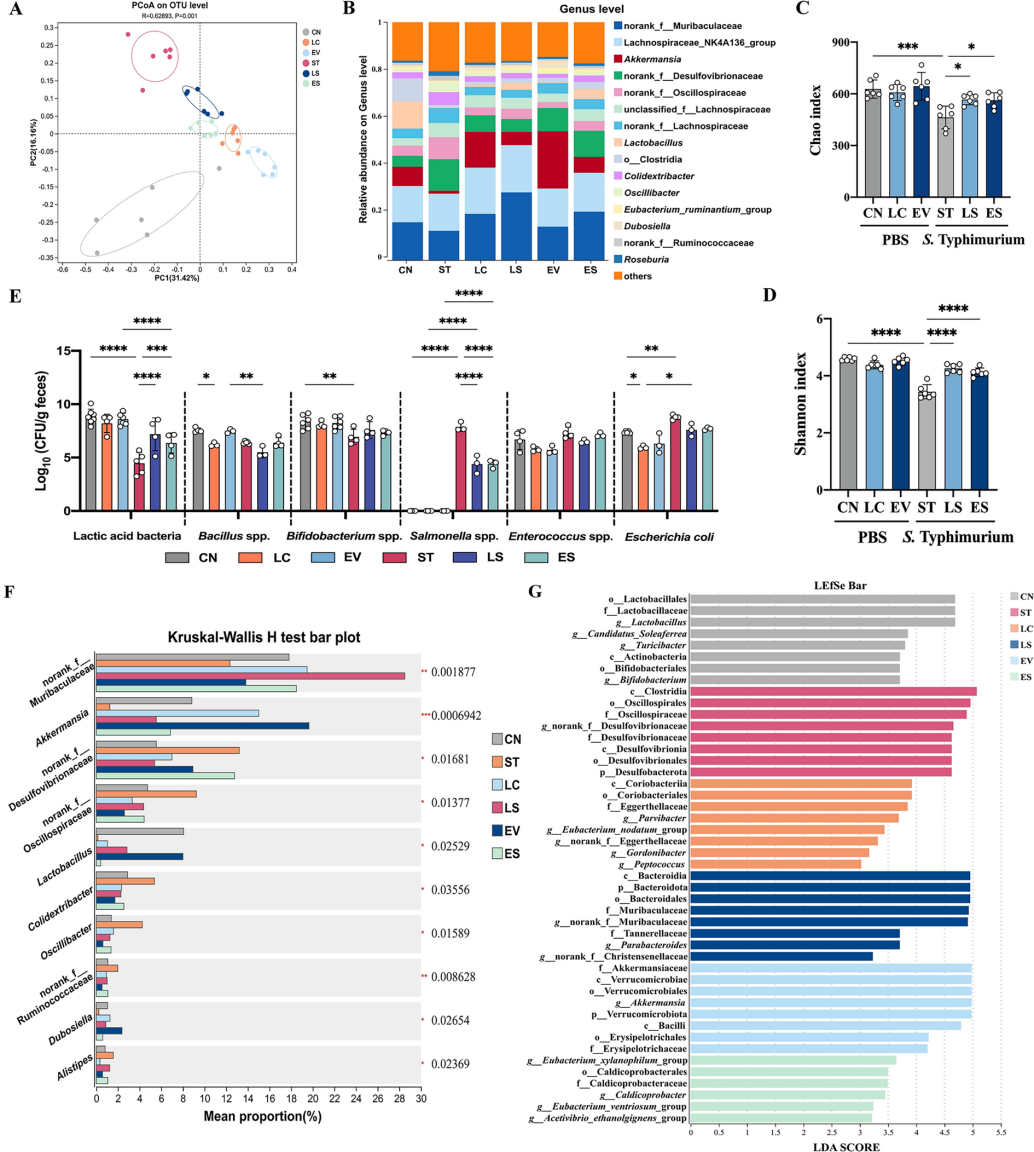
**Lj-EVs protect against *Salmonella* Typhimurium infection-induced intestinal microbiota dysbiosis.** (**A**) Principal coordinate analysis (PCoA) of β-diversity at the operational taxonomic unit (OTU) level, calculated using unweighted Unifrac distances. Data were generated from 16*S* rRNA gene amplicon sequencing (V3-V4 region, Illumina MiSeq) and normalized with QIIME 2. (**B**) Relative abundances of bacterial genus level in mouse gut microbiomes, determined by 16*S* rRNA sequencing. (**C–D**) Alpha-diversity metrics including Chao1 index (**C**) and Shannon index (**D**), calculated from rarefied OTU tables (10,000 reads per sample). (**E**) Quantification of fecal bacteria (Lactic acid bacteria, *Bacillus* spp., *Bifidobacterium* spp., *Salmonella* spp., *Enterococcus* spp. and *Escherichia coli*) of mice were determined by plate culture method. Data are shown as colony-forming units (CFU) per gram of feces, with individual data points overlaid on mean ± SD. (**F**) Group comparisons of α-diversity indices using the Kruskal–Wallis test (nonparametric ANOVA). Bars represent median values; whiskers denote 95% confidence intervals. (**G**) Linear discriminant analysis (LDA) effect size (LEfSe) results identifying differentially abundant taxa between groups. Bars indicate LDA scores (≥ 2.0,* P* < 0.05). All experiments included 6 biological replicates per group, except Fig.  4 d, which had *n* = 3–8 per group. Statistical significance was determined using two-tailed Mann–Whitney *U* test. Data represent the mean ± SD. For box-and-whiskers plots, the center line represents the median, the bounds of the box represent quartiles and the whiskers represent min to max. Statistically significant differences are shown with asterisks as follows: ^*^P < 0.05, ^**^P < 0.01, ^***^P < 0.001, ^****^P < 0.0001

To analyze the regulatory mechanism of Lj-EVs on the intestinal microbiota-barrier interaction, we performed 16*S* rRNA sequencing on mouse cecal contents. Principal coordinates analysis (PCoA) of β-diversity revealed a clear segregation of microbial communities among the groups, indicating significant differences in species composition (Fig. 4a). The community composition analysis showed that norank_f_Muribaculaceae (mucus secretion related [24]), Lachnospiraceae_NK4A136_group (carbohydrate metabolism [25]), and *Akkermansia* (barrier-enhancing bacteria [26]) were the dominant genera (Fig. 4b). *S*. Typhimurium infection (ST group) led to a decrease in the abundance of norank_f_Muribaculaceae and *Akkermansia* (*vs*. CN group), while Lj-EVs pretreatment restored them to nearly normal levels. The relative abundance of probiotics such as *Lactobacillus* in ES group was significantly higher than that of the ST group. In addition, the abundance of norank_f_Desulfovibrionaceae and norank_f_Oscillospiraceae was significantly increased in gut of mice infected with *S*. Typhimurium, which was reversed by pretreatment with either *L. johnsonii* or Lj-EVs (Fig. 4B). Further α-diversity analysis showed that Chao index (species richness) and Shannon index (diversity) in the ST group decreased (*P* < 0.01, Fig. 4c–d), while they significantly rebounded after Lj-EVs intervention. Consistent with this interaction, we also detected reduced levels of lactic acid bacteria and *Bifidobacterium* spp., and increased levels of *Escherichia coli* in fecal samples from the ST group during *Salmonella* infection, while levels were unchanged in the LS and ES groups (*P* < 0.01, Fig. 4e). Lj-EVs increased the relative abundance of *Lactobacillus* and reduced *Salmonella* colonization.

The intergroup abundance differences of key bacterial genera were evaluated by the Kruskal–Wallis H test (Fig. 4f). The abundance of mucus-degrading bacteria *Akkermansia* was significantly decreased in the *S*. Typhimurium infection group (ST group), while Lj-EVs intervention could increase the level in the cecum (*P* < 0.001). The abundance of norank_f_Muribaculaceae in ST group was increased significantly, which could be restored to normal level after Lj-EVs treatment (*P* < 0.01). Linear discriminant analysis of effect size (LEfSe) was used to detect the dominant difference of bacterial communities in each group (Fig. 4g). At the genus level, LEfSe analysis showed specific enrichment of the genera *g_Lactobacillus*, *g_Candidatus_Soleaferrea*, *g_Turicibacter*, c_Actinobacteria, and *g_Bifidobacterium* in CN group. In ST group, c_Clostridia, f_Oscillospiraceae, and p_Desulfobacterota were dominant. The p_Verrucomicrobiota and *g_Akkermansia* were dominant in EV group, while *g_Eubacterium_xylanophilum_group* was dominant in ES group. The o_*Bacteroidales* and f_Muribaculaceae were dominant in LS group (Fig. 4g, LDA score > 3.5 and *P* < 0.05). These results were consistent with the relative abundance analysis of genera in Fig. 4b. Collectively, these results indicated that Lj-EVs intervention remodeled the gut microbiota structure following *S*. Typhimurium infection, as characterized by increased abundance of beneficial and mucin-related bacteria. This reorganization of the microbial community was most likely associated with the observed amelioration in intestinal pathology and suggested a potential mechanism by which Lj-EVs might contribute to restore the intestinal barrier integrity.

### Lj-EVs remodeled the OXPHOS-glycolytic metabolic axis associated with changes in gut microbiota

**Figure 5:**
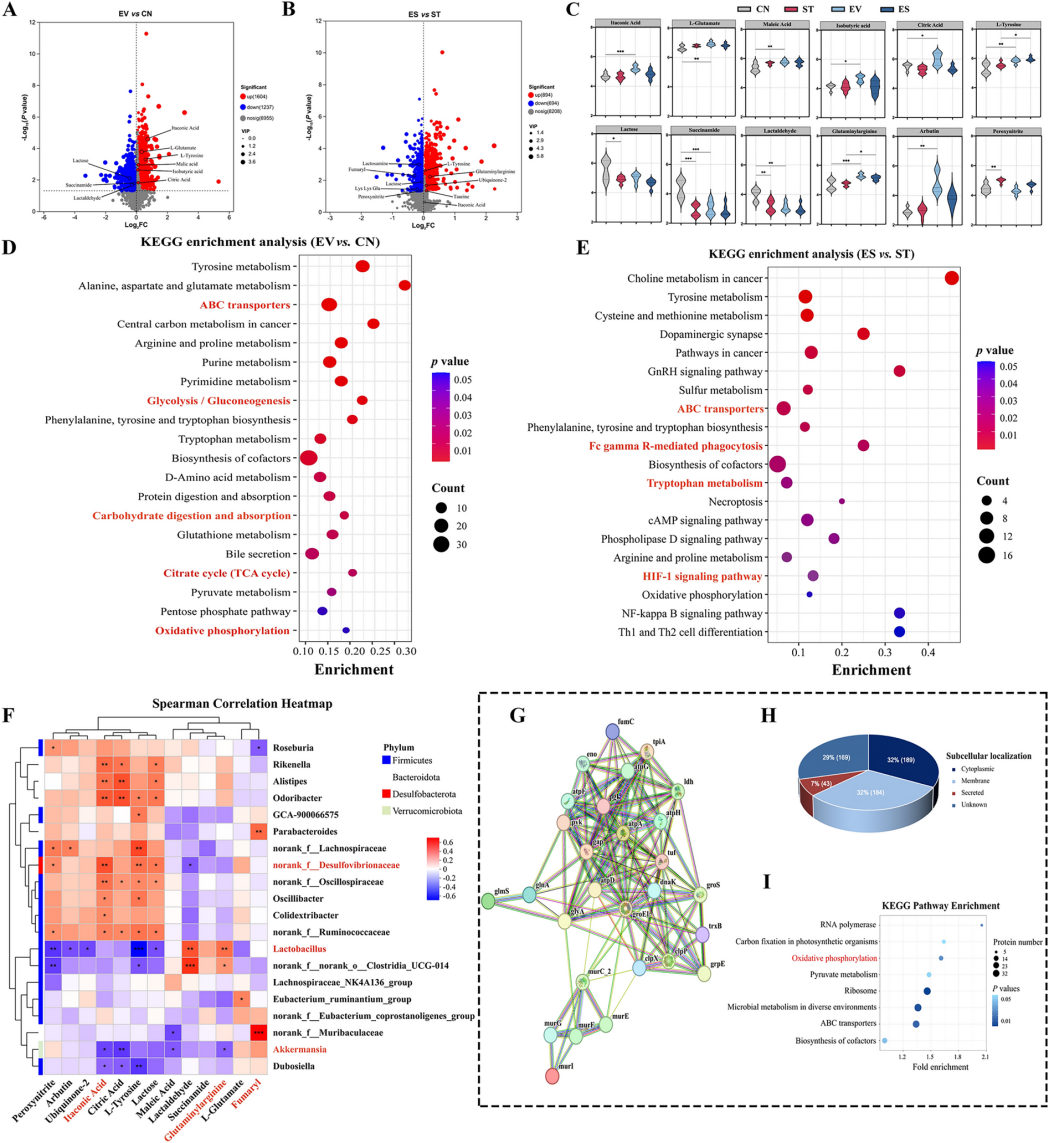
**Lj-EVs drive key metabolites of gut microbiota to restore oxidative phosphorylation and suppress glycolysis.** (**A**) Volcano plot of differentially expressed metabolites between EV and CN groups, identified by Welch’s *t*-test with Benjamini–Hochberg correction. Red points denote significantly up-regulated metabolites; blue points denote down-regulated metabolites. (**B**) Volcano plot comparing ES and ST groups, using identical statistical thresholds as in (**A**). (**C**) Violin plots of 12 key differentially expressed metabolites across all groups. Significance from Kruskal–Wallis test with Dunn’s post hoc correction. (**D**–**E**) KEGG pathway enrichment analysis of differentially expressed metabolites, showing top 30 enriched pathways. Bars represent −log_10_ (FDR), with pathway names ordered by significance. (**F**) Microbiota-metabolite crosstalk network, constructed via Spearman’s rank correlation (|ρ|≥ 0.5, *P* < 0.05). Nodes represent positive (red) or negative (blue) correlations. (**G**) Protein–protein interaction (PPI) network of the “oxidative phosphorylation-glycolysis” (oxphos-gly) cluster, generated via STRING v12.0 (confidence score ≥ 0.7) and visualized in Cytoscape v3.10. Nodes represent proteins; edges denote interactions. (**H**) Subcellular localization of Lj-EVs-associated proteins identified by liquid chromatography-tandem mass spectrometry (LC–MS/MS). Proteins were annotated against the *Lactobacillus johnsonii* UP000009078 proteome database (UniProt, release 2024_01), with subcellular compartments classified using CellOrganizer v2.1. A total of 585 unique proteins were identified through three repeated experiments. (**I**) KEGG pathway enrichment of Lj-EVs-associated proteins, showing significant enriched pathways (*P* < 0.05). Bubble size represents protein count; color intensity reflects *P* value. Statistically significant differences are shown with asterisks as follows: ^*^P < 0.05, ^**^P < 0.01, ^***^P < 0.001.

To investigate the impact of Lj-EVs on gut microbiota metabolism, we performed untargeted metabolomic analysis of mouse cecal contents. As shown in Additional file 3, principal component analysis (PCA) revealed distinct clustering of metabolic profiles among groups (*P* < 0.01, PERMANOVA). Volcano plot analysis (Fig. 5a) identified significantly altered metabolites (FDR < 0.05, fold change > 2) in EV group *vs*. CN group comparisons, with up-regulation of oxidative phosphorylation (OXPHOS) or anti-inflammatory—related metabolites (*e.g.*, maleic acid, citric acid, isobutyric acid, itaconic acid, L-Glutamate, and L-Tyrosine) and down-regulation of glycolysis or proinflammatory intermediates (*e.g.*, lactaldehyde, succinamide, and lactose). Conversely, ES group *vs*. ST group comparisons showed reversed trends: OXPHOS or anti-inflammatory-related metabolites (glutaminylarginine, ubiquinone-2, L-Tyrosine, and taurine) were elevated, while glycolytic or proinflammatory intermediates (lactosamine, peroxynitrite, fumaryl, and lys lys glu) were suppressed (Fig. 5b). Violin plots of 12 key metabolites in all group also demonstrated that Lj-EVs pretreatment alone elevated itaconic acid, L-Glutamate, maleic acid, citric acid, isobutyric acid, L-Tyrosine, glutaminylarginine, and arbutin (*P* < 0.05 or *P* < 0.01 *vs.* CN group; Fig. 5c), and ES mice elevated L-Tyrosine and glutaminylarginine (*P* < 0.05 *vs*. ST group; Fig. 5c). ROC analysis indicated the potential diagnostic value of these metabolites (AUC > 0.7, Additional file 3 C).

Pathway enrichment analysis further revealed that “Glycolysis/Gluconeogenesis”, “Citrate cycle (TCA cycle)”, and “Oxidative phosphorylation” as the enriched pathways in EV group *vs*. CN group (*P* < 0.01, Fig. 5d), while “HIF-1α signaling pathway” dominated ES group *vs*. ST group comparisons (*P* < 0.01, Figure 5E). Correlation heatmap analysis of the key metabolism-microbiota interaction network (Fig. 5f) revealed that Lj-EVs treatment reshaped specific microbe-metabolite associations. OXPHOS-glycolytic related metabolites (e.g., peroxynitrite, L-Tyrosine, itaconic acid, citric acid, and lactose) were significantly correlated with probiotic genera (norank_f_Desulfovibrionaceae, *Akkermansia,* and *Lactobacillus*) (|*P*|< 0.05), among which L-Tyrosine and itaconic acid showed the most significant correlations with them (28 and 22, respectively). Notably, itaconic acid exhibited a strong association with probiotic genera including *Akkermansia*. Given that itaconic acid possesses antimicrobial and anti-inflammatory properties via inhibition of succinate dehydrogenase and NLRP3 inflammasome [27], this association suggested a mechanistic link between Lj-EVs-driven probiotic genera enrichment and the observed immunometabolic modulation. Glutaminylarginine displayed strong positive correlations with both *Lactobacillus* and *Akkermansia*, potentially reflecting enhanced protein turnover and arginine metabolism in the remodeled mucosal niche. Fumaryl, associated with tyrosine catabolism and the TCA cycle [28], were predominantly correlated with norank_f_Muribaculaceae and *Parabacteroides*. In addition, our proteomic analysis identified the presence of fumarate hydratase class II within Lj-EVs, suggesting that Lj-EVs might directly contribute to fumarate metabolism and OXPHOS enhancement. Collectively, these correlation patterns highlighted a multi-dimensional crosstalk wherein Lj-EVs remodel specific microbial landscape that were functionally linked to metabolites involved in OXPHOS and anti-inflammatory pathways.

Finally, we observed differing metabolic regulation patterns between Lj-EVs and the parental *L. johnsoni*i probiotic. The Lj-EVs intervention was primarily associated with the OXPHOS-glycolysis metabolic axis (Fig. 5d–e), but the intact probiotic was mainly enriched in amino acid metabolic pathways such as arginine biosynthesis (Additional file 4). This indicated that Lj-EVs, as derivatives of their parent probiotics, might exert protective effects through mechanisms distinct from those of live bacteria, potentially involving multifaceted interactions between metabolic reprogramming and microbial ecology.

### Proteomic analysis of Lj-EVs revealed enrichment of metabolic and immunomodulatory proteins

To investigate whether Lj-EVs possess bioactive proteins capable of involving in the gut microbiota function, we conducted a comprehensive proteomic analysis using liquid chromatography-tandem mass spectrometry (LC–MS/MS). The identified peptides were compared and annotated according to the *Lactobacillus johnsonii* proteome database on UniProt, and a total of 585 proteins were identified through three repeated experiments. Details of the identified proteins were shown in Additional file 5. The subcellular structures of the identified proteins were predicted and classified statistically, and the results of vesicle proteomics data (Figure 5H) identified cytoplasmic proteins (32%) and membrane proteins (32%) as dominant components. Gene Ontology (GO) enrichment analysis (Additional file 6 A) demonstrated that the identified proteins in Lj-EVs were highly associated with critical biological processes (e.g., cellular metabolic processes, ATP synthesis), molecular functions (e.g., NAD(P)H binding, oxidoreductase activity), and cellular components (*e.g.*, membrane, ATP synthase complex). KEGG pathway analysis (Fig. 5i) further identified oxidative phosphorylation (*P* < 0.05) as a significantly enriched pathway, corroborated by domain enrichment analysis (Additional file 6) showing prominent NAD(P)H-binding domains and lactate/malate dehydrogenase motifs.

Protein–protein interaction (PPI) network analysis (Figure 5g) revealed a core functional complex comprising ATP synthase subunits (*e.g.*, atpA, atpD), TCA cycle regulators (*e.g.*, frdC, fumC, gabD), and Glycolytic enzymes (*e.g.*, eno, gap, pfk). The presence of this coordinated protein network suggested that Lj-EVs possess an intrinsic molecular mechanism to potentially modulate host metabolic pathways. In addition, we identified multiple immunomodulatory homologs that had been verified in other probiotics or symbiotic bacteria, such as fumarate hydratase class II (TCA cycle regulation), thioredoxin reductase (redox homeostasis), and tyrosine-protein phosphatase (inflammatory signaling) (mechanistic details in Additional file 7). These findings indicated that Lj-EVs were enriched in proteins with known functions in collectively mediate anti-inflammatory responses, microbial-epithelial crosstalk, and metabolic reprogramming.

### Lj-EVs modulated the expression of HIF-1α-glycolysis-related pathway and macrophage-related markers in intestinal tissues

**Figure 6:**
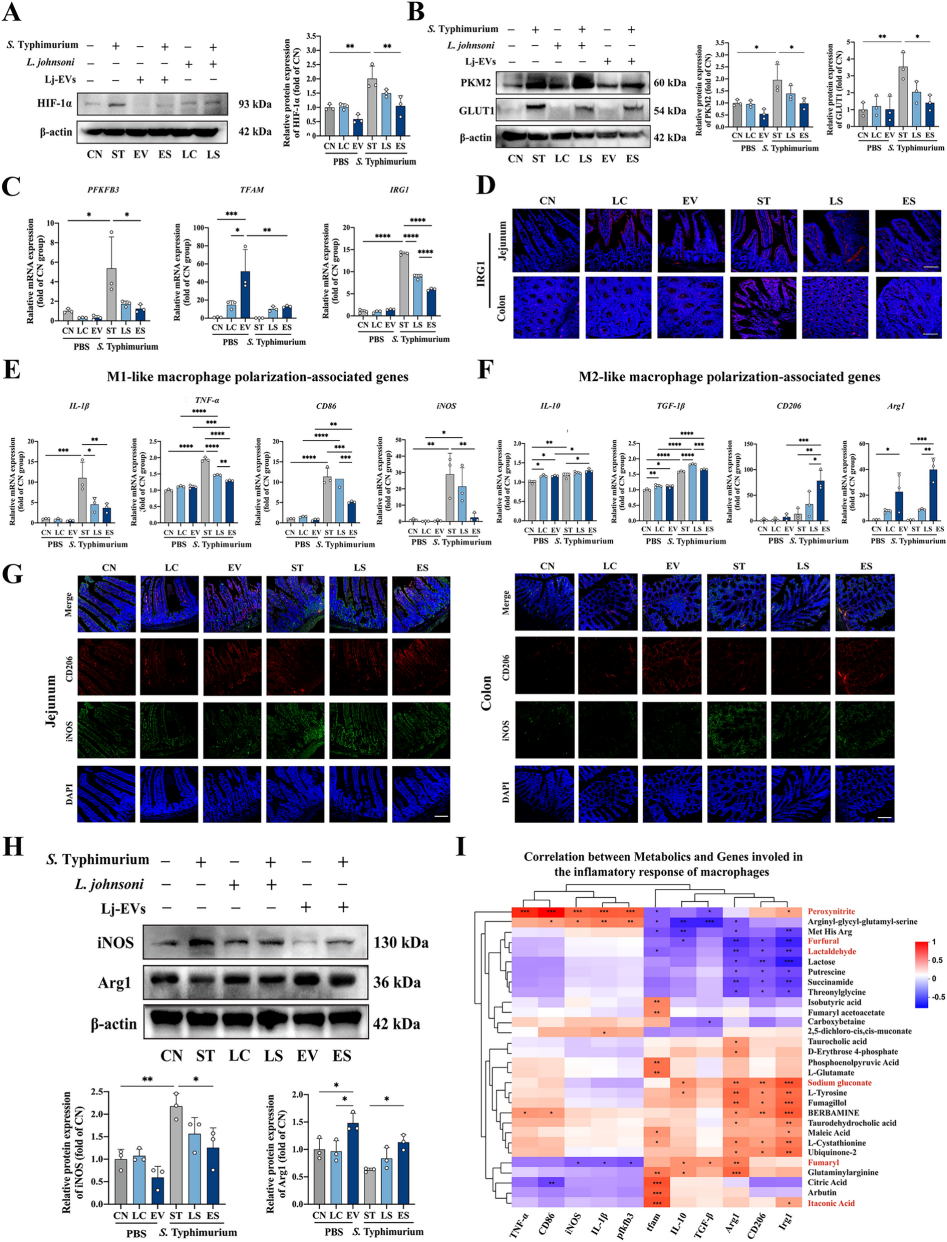
**Lj-EVs down-regulate the HIF-1α-glycolysis pathway and increase the expression of M1/M2 macrophage markers and metabolic genes in the jejunum tissue.** (**A**) Western blot analysis of HIF-1α protein expression in intestinal tissues with β-actin as a normalization control. (**B**) Western blot analysis of glycolysis-related proteins (PKM2, GLUT1) expression in intestinal tissues with β-actin as a normalization control. Data were normalized to β-actin and presented as mean ± SD from three independent biological replicates. Densitometry was performed using ImageJ. (**C**) Quantitative real-time PCR (qRT-PCR) analysis of oxidative phosphorylation metabolic marker gene (*PFKFB3*), glycolysis related marker gene (*TFAM*) and *IRG1* gene in jejunum of mice. Data were normalized to *β-actin* expression using 2^−ΔΔCt^ method and presented as means ± SD. Statistical significance determined by one-way ANOVA followed by Tukey’s multiple comparisons (*n* = 3 biologically replicates per group). (**D**) IF staining of *IRG1* (red) in jejunal and colonic lesions. Scale bars = 100 μm. (**E**–**F**) qRT-PCR analysis of (**E**) M1-like macrophage polarization-associated genes (*IL-1β, TNF-α, CD86,* and *iNOS*) and (**F**) M2-like macrophage polarization-associated genes (*IL-10, TGF-β, Arg1,* and *CD206*) in jejunum of mice, using same statistical methods as in (**C**). (**G**) IF staining of pro-inflammatory marker iNOS (green) and anti-inflammatory marker CD206 (red) in jejunal and colonic lesions. Scale bars = 100 μm. (**H**) Western blot analysis of macrophage polarization-associated proteins (iNOS, Arg1) expression in intestinal tissues with β-actin as a normalization control. (**I**) Pearson correlation matrix between metabolics and genes involved in the inflammation response of macrophages. Significance calculated using two-tailed test (|r|≥ 0.5, *P* < 0.05). Color intensity represents correlation coefficients; asterisks denote significance. Statistically significant differences are shown with asterisks as follows: ^*^P < 0.05, ^**^P < 0.01, ^***^P < 0.001, ^****^P < 0.0001.

Given the key regulatory role of the hypoxia-inducible factor 1α (HIF-1α) signaling pathway in metabolic regulation and inflammatory response of intestinal cells [29], we investigated whether Lj-EVs were associated with changes in this pathway. Western blot results showed that *S*. Typhimurium infection significantly up-regulated HIF-1α protein expression in mouse intestinal tissue, whereas Lj-EVs pretreatment restored it to near-basal level (Figure 6a) P < 0.01). Concordant with HIF-1αstabilization, infection up-regulated key glycolytic enzymes GLUT1 and PKM2. Lj-EVs pretreatment was associated with a significant reduction in the protein expression of both GLUT1 and PKM2 (Figure 6b)*P* < 0.05). At the transcriptional level, qPCR results showed that *S*. Typhimurium infection significantly increased the expression of *PFKFB3*, a key glycolytic gene, and this increase was attenuated by Lj-EVs pretreatment (Figure 6C). In addition, we observed that Lj-EVs significantly up-regulated the expression of mitochondrial transcription factor A (TFAM), a core regulator of oxidative phosphorylation, in the absence of infection (Figure 6C). These data supported the notion that the possible function of Lj-EVs was to participate in reshaping host cell metabolism, at least in part. We also examined the expression of immune response gene 1 (IRG1), a key node linking metabolism and immunity. Lj-EVs significantly inhibited infection-induced up-regulation of *IRG1* at the mRNA level (Figure 6C). This decrease in IRG1 protein expression in the jejunum and colon of the ES group was further confirmed by immunofluorescence (Figure 6D).

As macrophages undergo metabolic reprogramming during inflammation [30], we next assessed the expression of macrophage-related markers. Results in Figures 6E and F revealed that the mRNA expression levels of M1-liked macrophage polarization-associated markers (*IL-1β*, *TNF-α*, *iNOS,* and *CD86*) were reduced whereas those of M2-liked macrophage polarization-associated markers (*IL-10*, *TGF-β*, *CD206,* and *Arg1*) were elevated in the Lj-EVs-treated mice relative to infected mice. Immunofluorescence staining supported these findings, showing a marked decrease in iNOS-positive cells and an increase in CD206-positive cells in the intestinal tissues of the ES group compared to ST group. Western blot analysis further demonstrated that Lj-EVs intervention significantly reduced the protein expression of the pro-inflammatory marker iNOS and increased the expression of the anti-inflammatory marker Arg1 in mouse intestinal tissues (Figure 6H).

To explore potential connections between metabolic reprogramming regulated by Lj-EVs and macrophage polarization, we conducted a correlation heat map analysis of genes related to macrophage polarization phenotypes and top 30 key metabolites of gut microbiota in mice (Figure 6I). The heatmap revealed distinct phenotypes driven by metabolites: peroxynitrite exhibited robust positive correlations with pro-inflammatory M1 markers (e.g., *TNF-ɑ*, *CD86*, *iNOS*, *IL-1β*; *P* < 0.001) and key glycolytic-related genes (e.g., *PFKFB3*; *P* < 0.001), whereas fumaryl showed inverse associations (e.g., *iNOS*, *IL-1β*, *PFKFB3*; *P* < 0.05). Notably, sodium gluconate and fumaryl emerged as key coordinators of M2 polarization, demonstrating strong positive correlations with *Arg1* (*P* < 0.01)*,* and *IL-10* (*P* < 0.05) mRNA expression, while lactaldehyde and furfural inversely correlated with these anti-inflammatory M2 markers (e.g., *Arg1*, *CD206*, *IRG1*; *P* < 0.05 or *P* < 0.01). *IRG1* and *TFAM* genes were significantly correlated with most of the differential metabolites (17 and 14 of the top 30 metabolites, respectively). These data established that Lj-EVs orchestrated macrophage functional plasticity through glycolysis-oxidative phosphorylation dual metabolic nodes.

## Discussion

When *Salmonella* Typhimurium invaded the organism, it destroyed the tight junction structure between epithelial cells, and then was taken up by macrophages and transported to the submucosa [31]. The mucus layer formed by mucin secreted by goblet cells prevents intestinal microbes from entering the intestinal epithelium and is the first line of defense against infection [32, 33]. Our findings indicate that Lj-EVs could expand the mucus secretion by increasing the number of goblet cells, strengthen the chemical defense of the mucus layer by up-regulating MUC2 protein expression, and safeguard the integrity of the physical barrier by enhancing ZO-1 and Occludin protein expression. These observations suggest that Lj-EVs contribute to a multi-layered defense system against intestinal pathogens.

Beyond direct effects on host tissue, Lj-EVs were associated with a restructuring of the gut microbiota that is consistent with enhanced barrier function. Specifically, Lj-EVs intervention enriched mucin-utilizing symbionts (e.g., *Akkermansia*, norank_f_Muribaculaceae), suppressed potential pathobionts (e.g., norank_f_Desulfovibrionaceae, norank_f_Oscillospiraceae), and promoted the colonization of recognized probiotics. This microbiota remodeling will further cause a shift in the metabolic landscape [4]. The remarkable change was that Lj-EVs promoted the accumulation of metabolites with immunomodulatory functions. For instance, Lj-EVs up-regulated itaconic acid, an OXPHOS-related metabolite.

Itaconic acid could exert anti-inflammatory effects by inhibiting the NLRP3 inflammasome and regulating macrophage polarization [27]. Macrophage polarization driven by microbiota metabolite reprogramming can achieve pathogen clearance and anti-inflammatory functions [34]. Lj-EVs affected phenotypic changes in intestinal macrophage profile, including a reduction in M1 markers (e.g., iNOS, CD86) and an increase in M2 markers (e.g., Arg1, CD206). This shift was concomitant with the inhibition of HIF-1α-dependent glycolysis and enhancement of OXPHOS. Lj-EVs significantly down-regulated the glycolytic gene *PFKFB3* and up-regulated *IRG1.* IRG1 is known to catalyze the production of itaconic acid, which can destabilize HIF-1α and maintain redox homeostasis [35, 36]. This dual regulation may disrupt the proinflammatory loop triggered by *S*. Typhimurium and support the M2 polarization state [37]. Notably, the accumulation of itaconic acid identified by metabolomic analysis coincided with the up-regulation of IRG1, providing orthogonal validation that Lj-EVs drive reprogramming to shift macrophages toward an anti-inflammatory phenotype.

Proteomic analysis revealed that Lj-EVs were enriched in fumarate hydratase class II. Its presence might promote a shift of metabolic patterns toward OXPHOS that underpins M2 macrophage polarization [38]. Besides, Lj-EVs identified multiple cargo proteins with direct immunomodulatory potential, such as tyrosine protein phosphatases and thioredoxin reductase. Tyrosine protein phosphatases has been confirmed to regulate inflammatory factors by deactivate NF-κB signaling [39], and thioredoxin reductase can resist oxidative stress by using electrons provided by NADPH [40]. A recent study demonstrated that *L. johnsonii*-derived EVs carrying glyceraldehyde-3-phosphate dehydrogenase (GAPDH) alleviate colitis by inhibiting the MAPK-STAT3 axis in macrophages [41]. This is consistent with our findings that the protein cargo of probiotic-derived EVs may have the potential to participate in immune regulation.

An intriguing observation is that Lj-EVs recapitulated and in some metrics exceeded the protective effects of their parent probiotics. It is critical for understanding how probiotics exert anti-inflammatory function without requiring live bacteria administration. Lj-EVs recapitulated major beneficial effects of *L. johnsonii,* namely enhancing barrier integrity and modulating immunity. The nanoscale size of Lj-EVs likely enhanced their bioavailability and intestinal permeation while protecting enzymatic cargo from degradation [42, 43]. This nanoscale advantage makes Lj-EVs a promising alternative to live probiotics, particularly in hosts with weakened immune systems. While our multi-omics and tissue-level analyses strongly suggested macrophage reprogramming as a key mechanism, definitive confirmation requires future studies to analyze this effect specifically in macrophages. Moreover, elucidating the functional contribution of specific protein cargos within Lj-EVs to the microbiota-host immune axis will be crucial for unlocking their full therapeutic potential [44].

**Figure 7:**
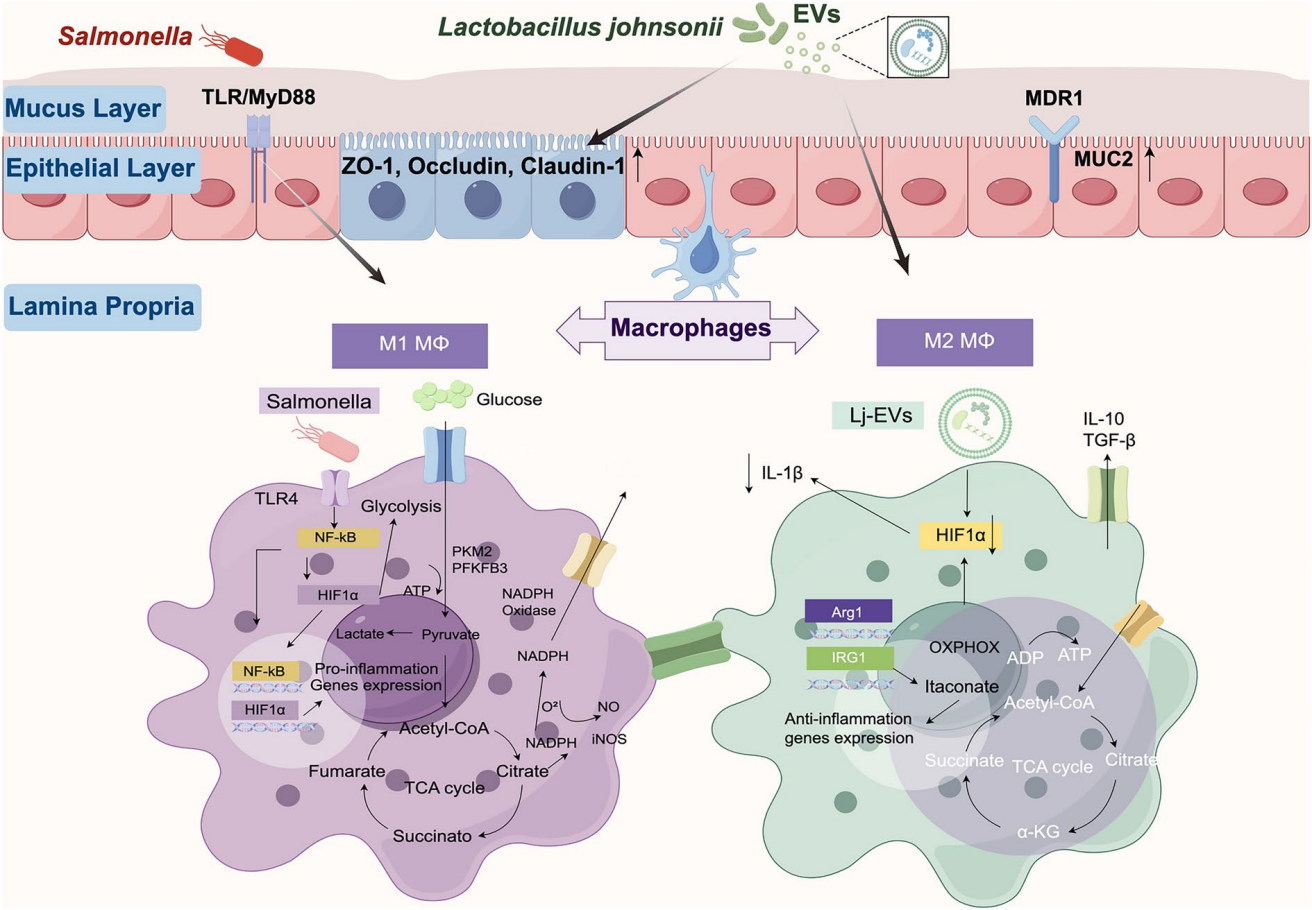
**Mechanism diagram of Lj-EVs ameliorating intestinal inflammation by mediating metabolic reprogramming of macrophages.** Figure 7 was created with Figdraw.com.

In conclusion, this study revealed that extracellular vesicles derived from *Lactobacillus johnsonii* enhance intestinal barrier function and host immunity and promote beneficial alterations of gut microbiota, which are associated with reprogramming of microbiota metabolites and ultimately mitigate infection-driven intestinal inflammation (Fig. 7). The multifaceted protective effects of Lj-EVs against *Salmonella* infection highlight their potential as a novel nanotherapeutic platform for infectious intestinal inflammation.

## Supplementary Information

**Additional file 1. Effects of *Lactobacillus johnsonii*-derived extracellular vesicles (Lj-EVs) on proliferation and inflammatory responses in LPS- or *Salmonella*-induced cell models.**

**Additional file 2. Lj-EVs improve the damage in liver and spleen of mice caused by** ***Salmonella* Typhimurium infection. **

**Additional file 3. The characteristics of Lj-EVs regulating the intestinal microbiota and its metabolites in mice. **

**Additional file 4. *Lactobacillus johnsonii* regulates intestinal metabolism in mice through multiple amino acid pathways.**

**Additional file 5. List of proteomic compositions of *Lactobacillus johnsonii*-derived extracellular vesicles related to Figure 5. **

**Additional file 6. Proteomics characterization of *Lactobacillus johnsonii*-derived extracellular vesicles (Lj-EVs).**

**Additional file 7. Information about the proteins identified in the Lj-EVs and their possible mechanisms mediating immune regulation.**

## Data Availability

The raw datasets of this article are available at the NCBI Sequence Read Archive (SRA) repository under accession number PRJNA1276970.

## Abbreviations

Lj-EVs: extracellular vesicles from *Lactobacillus johnsonii*
*S*. Typhimurium: *Salmonella* Typhimurium
*L. johnsonii*: *Lactobacillus johnsonii*
T3SS: type Ⅲ secretion system
SCVS: *Salmonella* Vesicles
TEM: transmission electron microscopy
NTA: nanoparticle tracking analysis
LC–MS/MS: liquid chromatography-tandem mass spectrometry
H&E: hematoxylin and eosin
AB-PAS: Alcian blue-periodic acid-Schiff
IF: immunofluorescence
IHC: immunohistochemical
BSA: bovine serum albumin
LPS: lipopolysaccharide
DAI: disease activity index
LEfSe: linear discriminant analysis of effect size
OXPHOS: oxidative phosphorylation
TCA cycle: Tricarboxylic Acid Cycle
HIF-1α: hypoxia-inducible factor 1α
TNF-α: Tumor Necrosis Factor alpha
IL-1β: interleukin-1 beta
iNOS: inducible Nitric Oxide Synthase
IRG1: immune response gene 1
TFAM: mitochondrial transcription factor A
PFKFB3: 6-phosphofructo-2-kinase/fructose-2,6-biphosphatase 3
Arg1: Arginase 1
