# Supplementary material for: Lactobacillus johnsonii-derived extracellular vesicles modulate gut microbiota metabolites and macrophage-related immune responses against Salmonella Typhimurium infection
Source: Vet Res. 2026 Apr 29;57:95. doi: 10.1186/s13567-026-01750-w (PMC13214080; doi:10.1186/s13567-026-01750-w)
Supplement: Supplementary file 7 — Additional file 7. Information about the proteins identified in the Lj-EVs and their possible mechanisms mediating immune regulation. [file 13567_2026_1750_MOESM7_ESM.docx]

**Additional file 7.** Information about the proteins identified in the Lj-EVs and their possible mechanisms mediating immune regulation.

| **Protein accession** | **Protein description** | **Gene name** | **Possible mechanisms that mediate immune regulation** |
| --- | --- | --- | --- |
| D0R3S0 | Fumarate hydratase class II | fumC | TCA cycle; degrades the pro-inflammatory metabolite fumarate to maintain bacterial metabolic fitness and promote pathogenesis |
| D0R548 | Thioredoxin reductase | trxB | Regulates reactive oxidative metabolism and the reduction/oxidation balance |
| D0R536 | ATP-dependent Clp protease proteolytic | clpP | Potential antimicrobial targets |
| D0R6A2 | ABC transporter, CydDC cysteine exporter (CydDC-E) family | cydC | ABC transporter (with CydD) that exports glutathione and cysteine to maintain redox homeostasis in the periplasm |
| D0R614 | Tyrosine-protein phosphatase | tpk | Activation and function of B cells |
| D0R3E0 | non-specific serine/threonine protein kinase | prkC | Regulated cell wall homeostasis, chain length, and biofilm formation |
| D0R2F3 | Chaperonin GroEL | groEL | Molecular chaperone essential for protein folding, assembly, and transport under stress conditions; immunomodulatory protein that influences dendritic cell responses |

The whole list of proteome composition, the INTERPRO analysis, and the KEGG analysis was introduced in additional file 5.
